# Effective treatment of mogamulizumab-induced head and neck dermatitis with fluconazole in a patient with peripheral T-Cell lymphoma

**DOI:** 10.1016/j.jdcr.2021.11.022

**Published:** 2021-12-15

**Authors:** Ishan Asokan, Sanminder Singh, John Moesch, Jonhan Ho, Oleg E. Akilov

**Affiliations:** a Department of Internal Medicine, University of California, Los Angeles, California; b Department of Dermatology, University of Pittsburgh, Pittsburgh, Pennsylvania

**Keywords:** head and neck dermatitis, mogamulizumab-kpkc, peripheral T-cell lymphoma, CCR4, chemokine receptor 4, MAR, mogamulizumab-kpkc–associated drug rash, PTCL, peripheral T-cell lymphoma, Treg cells, T regulatory cells

Mogamulizumab-kpkc is a humanized defucosylated monoclonal antibody that targets transmembrane chemokine receptor 4 (CCR4). Aberrant CCR4 expression occurs in lymphoproliferative disorders, particularly in cutaneous and peripheral T-cell lymphomas (PTCL), and is associated with advanced disease and worse prognosis.^1^ Mogamulizumab-kpkc serves as a novel tool in treating advanced T-cell lymphomas, with a 34% overall response rate in a Japanese phase II study of 29 patients with PTCL.^2^ There is, however, mounting evidence of mogamulizumab-kpkc–related cutaneous eruptions. In addition to malignant lymphocytes, other skin-resident T lymphocytes that express CCR4 highly, such as naive activated CD4^+^ T cells, memory CD4 T-helper type 2 cells, memory T regulatory (Treg) cells, memory CD4^+^ T-helper type 17 cells, memory CD4^+^ T cells with follicular helper phenotype, and, to a lesser extent, naive Treg and naive activated CD8^+^ T cells,^3^ may be targets for mogamulizumab-kpkc. Depletion of CCR4^+^ Treg cells by mogamulizumab-kpkc not only may augment antitumor immunity, as was shown recently,^4^ but also may elicit cutaneous inflammatory responses, as was shown in Foxp3^KO^ CCR4^KO^ murine chimeric models.^5^ We observed this ambivalent effect of mogamulizumab-kpkc in a patient with lymphoma and discussed the appropriate management.

## Case report

**Fig 1:**
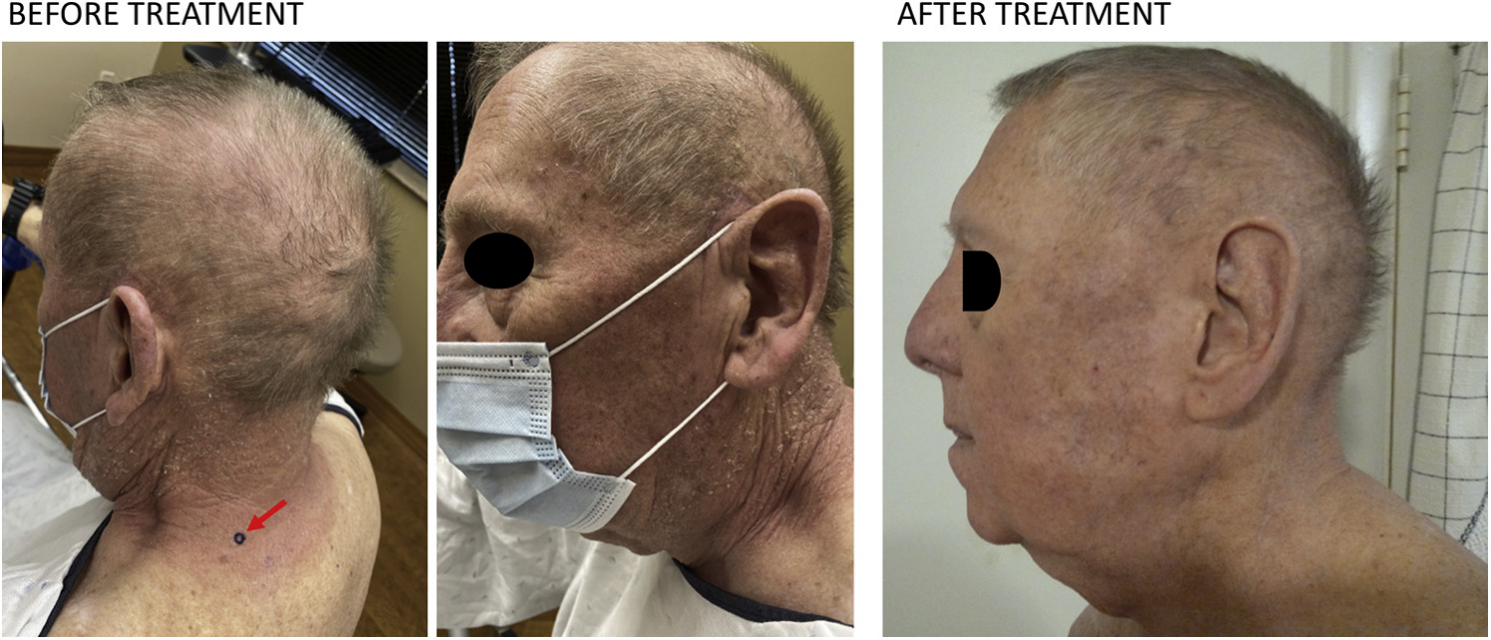
Before treatment, clinical examination shows confluent erythematous patches and eczematous plaques with overlying greasy scale on the head and neck (*arrow* indicated the place of biopsy). After treatment, there is complete resolution of erythematous patches on the head and neck area without the need for continued topical and systemic antifungal therapy.

**Fig 2:**
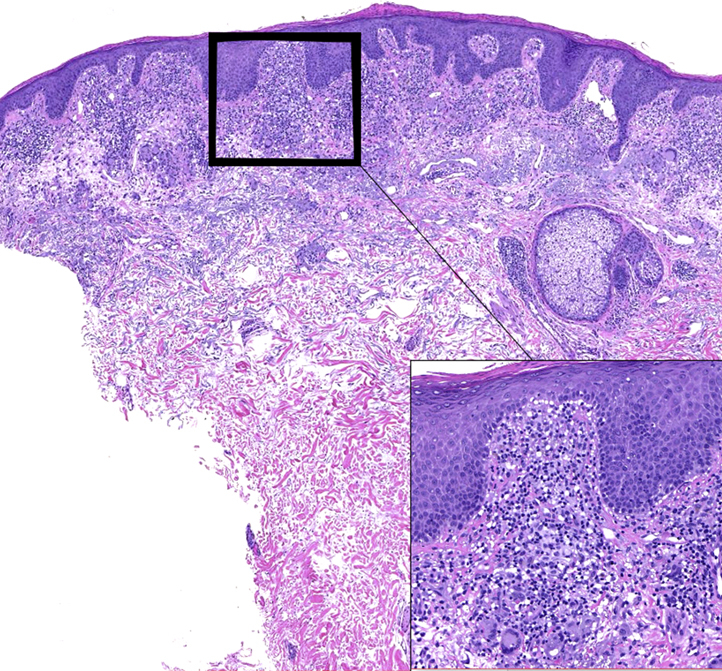
Lichenoid and granulomatous inflammation with hyperparakeratosis, hypergranulosis, irregular acanthosis, and degeneration of basal keratinocytes. (Hematoxylin-eosin stain; original magnification: ×10.) *Inset*: the dermis demonstrates a bandlike superficial infiltrate composed predominantly of lymphocytes, histiocytes, epithelioid histiocytes, multinucleated giant cells, eosinophils, and occasional melanophages. (Hematoxylin-eosin stain; original magnification: ×40.)

A 76-year-old man with a history of resected renal cell carcinoma presented with peripheral CD4^+^ T-cell lymphoma, not otherwise specified, that was diagnosed 6 years previously. The patient had only mild generalized lymphadenopathy without splenic or marrow infiltration. After completion of 3 cycles of prednisone, cyclophosphamide, vincristine, and doxorubicin, his lymphadenopathy resolved, and he had only cutaneous involvement. After treatment with romidepsin, he had partial resolution, but a relapse of PTCL occurred 2 years later. He completed 4 cycles of liposomal doxorubicin, resulting in remission for 2 more years. After another recurrence of lymphoma in the skin only, the patient received mogamulizumab-kpkc in combination with pegylated interferon alfa-2a every 2 weeks. Because of intolerance of pegylated interferon, the patient was transitioned to monotherapy with mogamulizumab-kpkc. Two years later, the patient presented with erythematous patches covered in greasy lamellar scale with secondary lichenification on both sides of his neck, consistent with head and neck dermatitis (Fig 1). A punch biopsy showed hyperparakeratosis, hypergranulosis, irregular acanthosis, and degeneration of basal keratinocytes with scattered dyskeratotic cells at the dermoepidermal junction (Fig 2). A granulomatous infiltrate composed of lymphocytes, histiocytes, epithelioid histiocytes, multinucleated giant cells, eosinophils, and occasional melanophages was consistent with a clinical impression of a drug-induced eruption.

Because of suspicion of a *Malassezia-*driven head and neck dermatitis in our patient, we initiated fluconazole 200 mg daily for 7 consecutive days per month for 2 months, along with ketoconazole shampoo/cream applied twice daily. On follow-up 8 weeks later, the patient had complete resolution of his head and neck dermatitis (Fig 1).

## Discussion

Mogamulizumab-kpkc, which was initially developed for treatment of adult T-cell leukemia and lymphoma, was approved by the Food and Drug Administration for treatment of relapsed and refractory PTCL and cutaneous T-cell lymphoma.^6^ With widespread use of mogamulizumab-kpkc, clinicians observed an array of adverse reactions related to its use, including mogamulizumab-kpkc–associated drug rashes (MARs).^7^

Despite the high frequency of MARs, there is limited consensus on an effective treatment approach, partly due to several diverging proposed mechanisms for the development of these MARs. One such mechanism involves activation of the immune system due to the depletion of Treg cells, leading to a graft versus host disease-type interface dermatitis,^8^ or the anti-CCR4 effects of mogamulizumab-kpkc, leading to polarization of T-helper type 1 cells and a granulomatous dermatitis.^9^ The common factor among these mechanisms is the role of mogamulizumab-kpkc in the depletion of Treg cells, leading to an aberrant immunologic environment. Resident Treg cells, which are found both in skin and in transformed tumor microenvironments in patients with cutaneous lymphoma,^10^ are a subset of CD4^+^ T cells that suppress pathogenic immune responses. Treg cells classically function to ensure tissue immune homeostasis and tolerance of commensal organisms both on the skin and in the enteric system.^11^ CCR4 and CCR10 are prominent chemokine receptors that facilitate transmigration of CD4^+^ Treg cells through the endothelium to the skin by binding to ligands CCL17, CCL22, and CCL27. When CCR4 blockade is selectively achieved by administration of mogamulizumab, there is impairment of global Treg function and development of a rebound inflammatory response against commensal organisms.^12^ There is a report of a patient in whom a severe, treatment-refractory colitis developed during treatment with mogamulizumab, which further highlights the impact of depletion of Treg cells and loss of tolerance of cutaneous and enteric commensal organisms.^13^

The MAR noted in our patient had a primarily granulomatous histologic pattern, with a clinical presentation resembling *Malassezia-*driven head and neck dermatitis, as observed in patients with severe atopic dermatitis. Itraconazole and azole antifungals were effective treatments in more than two thirds of adult patients with head and neck dermatitis on atopic skin background^14^ and also were found to be effective in our patient.

In conclusion, patients treated with mogamulizumab-kpkc may be at risk for developing a counterreactive immune response caused by depletion of Treg cells, which normally provide tolerance to commensal organisms. The conversely reactive inflammatory state manifests in a number of MARs with various histologic subtypes,^7^ such as the lichenoid or granulomatous dermatitis seen in our patient. Failure to respond to conventional topical therapies, such as steroids or emollients, also alerts the clinician to suspect an underlying yeast etiology. Timely recognition and appropriate treatment avoid interruption of biologic therapy for underlying lymphoma and improve the overall quality of life of these patients.

## Conflicts of interest

None disclosed.
