# Correction

**DOI:** 10.1016/j.jdcr.2022.02.012

**Published:** 2022-02-14

**Asokan I, Singh S, Moesch J, Akilov O E. Effective treatment of mogamulizumab-induced head and neck dermatitis with fluconazole in a patient with peripheral T-Cell lymphoma. *JAAD Case Reports.* 2022; 20:44-46.**

In the original publication, there was a typo within the title, this error has been corrected online.

